# Factors Affecting Non-Enzymatic Protein Acylation by *trans*-3-Methylglutaconyl Coenzyme A

**DOI:** 10.3390/metabo14080421

**Published:** 2024-07-31

**Authors:** Elizabeth A. Jennings, Megan M. Macdonald, Irina Romenskaia, Hao Yang, Grant A. Mitchell, Robert O. Ryan

**Affiliations:** 1 Department of Biochemistry and Molecular Biology, University of Nevada, Reno, NV 89557, USA; elizabethjennings@unr.edu (E.A.J.); meganmacdonald@unr.edu (M.M.M.); irina@unr.edu (I.R.); 2 Medical Genetics Service, Department of Pediatrics and Research Center, CHU Sainte-Justine and Université de Montréal, Montreal, QC H3T 1C5, Canada; hao.yang.hsj@ssss.gouv.qc.ca (H.Y.); grant.mitchell.med@ssss.gouv.qc.ca (G.A.M.)

**Keywords:** 3-methylglutaconyl CoA, protein acylation, inborn errors of metabolism, HMG CoA lyase deficiency, immunoblot, primary 3MGC aciduria

## Abstract

The leucine catabolism pathway intermediate, *trans*-3-methylglutaconyl (3MGC) CoA, is considered to be the precursor of 3MGC acid, a urinary organic acid associated with specific inborn errors of metabolism (IEM). *trans*-3MGC CoA is an unstable molecule that can undergo a sequence of non-enzymatic chemical reactions that lead to either 3MGC acid or protein 3MGCylation. Herein, the susceptibility of *trans*-3MGC CoA to protein 3MGCylation was investigated. *trans*-3MGC CoA was generated through the activity of recombinant 3-methylcrotonyl CoA carboxylase (3MCCCase). Following enzyme incubations, reaction mixtures were spin-filtered to remove 3MCCCase. The recovered filtrates, containing *trans*-3MGC CoA, were then incubated in the presence of bovine serum albumin (BSA). Following this, sample aliquots were subjected to α-3MGC IgG immunoblot analysis to probe for 3MGCylated BSA. Experiments revealed a positive correlation between *trans*-3MGC CoA incubation temperature and 3MGCylated BSA immunoblot signal intensity. A similar correlation was observed between incubation time and 3MGCylated BSA immunoblot signal intensity. When *trans*-3MGC CoA hydratase (AUH) was included in incubations containing *trans*-3MGC CoA and BSA, 3MGCylated BSA immunoblot signal intensity decreased. Evidence that protein 3MGCylation occurs in vivo was obtained in studies with liver-specific 3-hydroxy-3-methylglutaryl (HMG) CoA lyase knockout mice. Therefore, *trans*-3MGC CoA is a reactive, potentially toxic metabolite, and under normal physiological conditions, lowering *trans*-3MGC CoA levels via AUH-mediated hydration to HMG CoA protects against aberrant non-enzymatic chemical reactions that lead to protein 3MGCylation and 3MGC acid production.

## 1. Introduction

**Figure 1:**
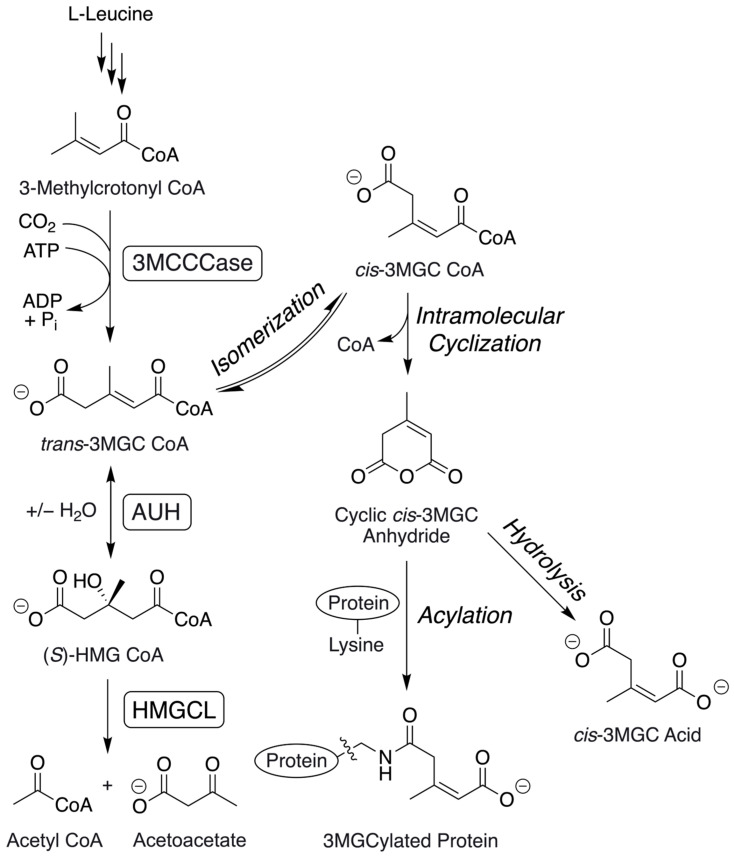
Alternate fates of *trans*-3MGC CoA. In the leucine degradation pathway, *trans*-3MGC CoA is generated as a pathway intermediate. Whereas this intermediate is normally metabolized to acetoacetate and acetyl CoA by AUH and HMGCL activities, respectively, an alternate non-enzymatic chemical reaction sequence (see italics) leads to protein 3MGCylation.

Inborn errors of metabolism (IEMs) in specific leucine catabolism pathway enzymes manifest 3-methylglutaconic (3MGC) aciduria as a phenotypic feature [1,2]. During leucine catabolism, the α-ß unsaturated acyl CoA intermediate, 3-methylcrotonyl (3MC) CoA, is converted to *trans*-3MGC CoA by 3MC CoA carboxylase (3MCCCase) (Figure 1). Following this, *trans*-3MGC CoA is converted to 3-hydroxy-3-methylglutaryl (HMG) CoA by *trans*-3MGC CoA hydratase (AUH). Finally, HMG CoA is converted to acetoacetate and acetyl CoA by HMG CoA lyase (HMGCL). When HMGCL or AUH are missing or deficient, large quantities of the organic acid, 3MGC acid, are excreted in urine [3,4]. In AUH deficiency, once *trans*-3MGC CoA is formed, it cannot proceed further down the leucine catabolism pathway, and because the reaction catalyzed by 3MCCCase is essentially irreversible [5], it cannot be metabolized back to 3MC CoA. In HMGCL deficiency, although the pathway impediment occurs at the level of HMG CoA because AUH catalyzes a reversible hydration/dehydration reaction [6], HMG CoA can be dehydrated back to *trans*-3MGC CoA. As *trans*-3MGC CoA is produced in these IEMs, it is susceptible to non-enzymatic chemical reactions that culminate in the formation of 3MGC acid or protein 3MGCylation [7]. In this reaction sequence, isomerization generates *cis*-3MGC CoA that is structurally poised to undergo intramolecular cyclization, yielding 3MGC anhydride and free CoA [8]. Once formed, the anhydride has at least two possible fates, including spontaneous hydrolysis to 3MGC acid or covalent 3MGCylation of protein lysine side chain amino groups [8,9]. In vivo, 3MGCylated proteins are substrates for the NAD^+^-dependent deacylase, sirtuin 4 [10,11]. This enzyme ultimately releases *cis*-3MGC acid, which is transported out of mitochondria and excreted in urine. Based on its intrinsic chemical properties, *trans*-3MGC CoA is a labile compound. Under normal physiological conditions, *trans*-3MGC CoA does not undergo aberrant non-enzymatic chemical reactions because it is efficiently converted to HMG CoA by AUH. Consistent with this, studies indicate that the equilibrium constant, k_eq_, for AUH strongly favors the hydration of *trans*-3MGC CoA over the dehydration of HMG CoA [12].

HMG CoA lyase (HMGCL) deficiency is a severe IEM that prevents leucine catabolism in all tissues and ketone body biosynthesis in the liver and kidneys. HMGCL deficiency is associated with hypoketotic hypoglycemia, hyperammonemia, metabolic acidosis, and potentially fatal cardiomyopathy of unknown pathophysiology [13,14]. This disorder has been modeled by liver-specific [15] and cardiomyocyte-specific [16] HMGCL gene ablation in mice. Liver-specific HMGCL knockout (KO) mice display elevated levels of HMG acid, 3-methylglutaric acid, and 3MGC acid in urine. Moreover, an increase in protein HMGylation was observed in HMGCL liver-specific KO mouse liver lysates as compared to control mice [17]. Liver-specific and heart-specific HMGCL KO mice also display increased levels of leucine-pathway acyl CoA intermediates (e.g., HMG CoA, 3MC CoA, and isovaleryl CoA) in liver and heart tissue, respectively. Levels of these acyl CoAs increase upon the administration of the upstream leucine degradation pathway intermediate, 2-ketoisocaproate (KIC) [15,16]. Therefore, these mice serve as a potential in vivo model of protein 3MGCylation induced by HMGCL deficiency.

In the present study, *trans*-3MGC CoA chemical reactivity was studied in vitro to evaluate factors that affect non-enzymatic chemical reactions that lead to covalent protein acylation. Since *trans*-3MGC CoA is not commercially available, recombinant 3MCCCase was employed in in vitro enzyme assays to produce *trans*-3MGC CoA [9,18]. Following the removal of 3MCCCase from the reaction product mixture, *trans*-3MGC CoA was subjected to secondary incubations in the presence of bovine serum albumin (BSA). Under the conditions employed, as *trans*-3MGC CoA is converted to 3MGC anhydride, it covalently acylates lysine side chain amino groups on BSA, a modification that is detectable using α-3MGC IgG immunoblot analysis. By using this strategy, evidence was obtained that the extent of *trans*-3MGC CoA-dependent BSA 3MGCylation positively correlates with incubation temperature and time. Moreover, results obtained reveal that when AUH is included in incubations containing *trans*-3MGC CoA and BSA, the amount of 3MGCylated BSA formed is reduced. To investigate protein 3MGCylation in vivo, comparative immunoblot analysis was performed on mitochondrial homogenates obtained from liver-specific HMGCL KO and control wild-type mice. Compared with wild-type mice, numerous HMGCL KO mouse liver mitochondrial proteins were positive for 3MGCylation. Moreover, upon administration of the leucine catabolism pathway intermediate, KIC, to these mice, increased protein 3MGCylation was observed. In this article, we show that *trans*-3MGC CoA is labile and, in IEMs of leucine degradation that involve steps downstream of *trans*-3MGC CoA, 3MGCylated proteins/3MGC acid are produced via a sequence of non-enzymatic chemical reactions.

## 2. Materials and Methods

### 2.1. Chemicals, Reagents, and Enzymes

3-methylcrotonyl (3MC) CoA was obtained from Sigma Chemical Co. (St. Louis, MO). *Xanthomonas citri* 3-methylcrotonyl CoA carboxylase (3MCCCase) was expressed in *E. coli* and isolated as described by Young et al. [9]. Recombinant *A. thaliana* AUH was expressed in *E. coli* and isolated as described previously [19]. BSA was obtained from Millipore-Sigma (Burlington, MA) and used without further modification. 3-methylglutaconic anhydride (4-methyl-2H-pyran-2,6(3H)-dione) was obtained from Biosynth Ltd.(Compton, UK), and 5,5’-dithiobis(2-nitrobenzoic acid; DTNB) was obtained from Sigma-Aldrich.

### 2.2. 3-Methylcrotonyl CoA Carboxylase (3MCCCase) Activity Assays

Isolated recombinant 3MCCCase (6.7 µg per 100 µL) was incubated in a buffer (50 mM HEPES, pH 8.0, 20 mM MgCl_2_, 20 mM KCl) containing 10 mM ATP, 10 mM NaHCO_3_, and 275 µM 3MC CoA [9]. Enzyme assays were conducted at 20 °C for 2 h and stopped by removing 3MCCCase through spin filtration (10 kDa molecular weight cutoff). SDS-PAGE analysis of the retentate and filtrate fractions confirmed that 3MCCCase was not present in the filtrate. The recovered filtrate, containing *trans*-3MGC CoA, was then used in incubations with BSA (0.5 mg/mL) under specified conditions, followed by the detection of 3MGCylated BSA using α-3MGC IgG immunoblot analysis [8]. By using this assay protocol, the effect of variation in filtrate incubation conditions was examined.

### 2.3. Effect of Temperature on 3MGC Anhydride Formation

Following the spin filtration of a 3MCCCase assay product mix and a control assay mix lacking 3MCCCase, aliquots of the respective filtrates were incubated for 4 h at 4, 20, 37, 55, and 70 °C, respectively. Following incubation, samples were assayed for the presence of free thiol (produced upon intramolecular cyclization). Individual sample aliquots (30 µL of a 200 µL total final reaction volume) were incubated with 100 µM DTNB in 100 mM sodium phosphate, pH 8.0, containing 1 mM EDTA for 15 min at room temperature, followed by the measurement of sample absorbance at 412 nm on a SpectraMax M5 microplate reader (Molecular Devices, San Jose, CA USA).

### 2.4. Effect of Incubation Temperature on 3MGC Anhydride Reactivity

3MGC anhydride was dissolved (275 µM) in 50 mM HEPES, pH 8.0, 20 mM MgCl_2_, and 20 mM KCl and incubated for 4 h at 4, 20, 37, 55, and 70 °C, respectively. Following incubation, each sample was adjusted to 20 °C and BSA (0.5 mg/mL) was added prior to a further 1 h incubation at 20 °C. Following this incubation, an aliquot of each sample was subjected to α-3MGC IgG immunoblot analysis to probe for 3MGCylated BSA.

### 2.5. Effect of Incubation Temperature and AUH on trans-3MGC CoA-Dependent Acylation of BSA

A 3MCCCase assay mix containing BSA (0.5 mg/mL) was incubated at specified temperatures (4, 30, 37, 45, and 55 °C, respectively) for 5 h in the presence or absence of AUH (2 µg per 100 µL). Control assays lacking 3MCCCase or 3MC CoA were performed in parallel and incubated at 37 °C. Following incubation, an aliquot of each sample was subjected to α-3MGC IgG immunoblot assay to measure the 3MGCylated BSA signal intensity.

### 2.6. Effect of Incubation Time and AUH on trans-3MGC CoA-Dependent Acylation of BSA

A 3MCCCase assay mixture containing BSA (0.5 mg/mL) was incubated in the presence or absence of AUH (2 µg per 100 µL). Samples were then incubated at 37 °C for 2, 4, 6, and 8 h, followed by α-3MGC IgG immunoblot detection of 3MGCylated BSA signal intensity. A control assay lacking 3MCCCase was incubated at 37 °C for 8 h prior to immunoblot analysis.

### 2.7. Liver-Specific HMGCL KO Mice Studies

HL^L/L^ mice, in which both alleles of *Hmgcl* exon 2 are flanked by LoxP sites and which manifest complete deficiency of HMG CoA lyase in the liver, were designed as previously described [15,16]. Where indicated, eight-week-old male liver-specific HMGCL KO mice and wild-type control mice were administered KIC (2 mg/g body weight) or saline via intraperitoneal injection as previously described [15]. Mouse livers were harvested, and mitochondria were isolated as previously described [20]. Total protein levels for wild-type and HMGCL KO mouse liver mitochondrial samples were measured using BCA assay, and equivalent sample protein aliquots were subjected to α-3MGC IgG immunoblot analysis. Ponceau staining was used as a loading control.

## 3. Results

### 3.1. Effect of Incubation Temperature and Time on trans-3MGC CoA-Dependent Acylation of BSA

**Figure 2:**
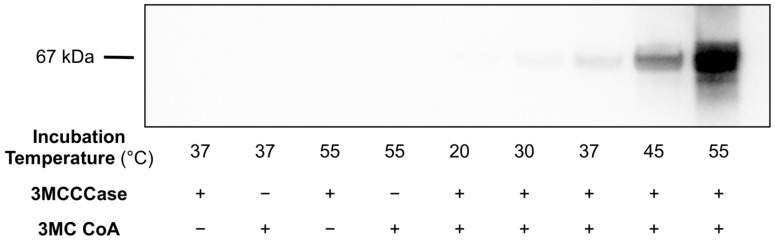
Effect of incubation temperature on *trans*-3MGC CoA-dependent acylation of BSA. A 3MCCCase enzyme assay was conducted and filtered to remove 3MCCCase. BSA (0.5 mg/mL) was added to the filtrate fraction, and sample aliquots were incubated for 4 h at temperatures ranging from 20 °C to 55 °C. For control incubations, the 3MCCCase assay was conducted in the absence of a 3MC CoA substrate or 3MCCCase enzyme. Following incubation, samples were probed for 3MGCylated BSA using α-3MGC IgG immunoblot analysis. The immunoblot shown is representative of an experiment performed on three separate occasions.

To determine the effect of incubation temperature on *trans*-3MGC CoA chemical reactivity, 3MCCCase assay filtrate aliquots were incubated with BSA (0.5 mg/mL) for 4 h at temperatures ranging from 20 °C to 55 °C. Following incubation, samples were analyzed using α-3MGC IgG immunoblot analysis to measure 3MGCylated BSA signal intensity (Figure 2). No signal was detected when either the 3MCCCase assay substrate, 3MC CoA, or the enzyme 3MCCCase were omitted from the initial incubation. When both substrate and enzyme were present, the resulting filtrate induced a temperature-dependent increase in BSA 3MGCylation. The signal at 55 °C was very strong such that, at immunoblot exposure times that produce a reasonable signal intensity, the corresponding signal intensities observed at 20 °C, 30 °C, and 37 °C, respectively, are faint. At longer exposure times, however, a strong temperature-dependent increase in signal intensity was observed between 20 °C and 37 °C (data not shown). It is noteworthy that relatively low levels of BSA 3MGCylation were observed when 3MCCCase assay filtrates were incubated with BSA for 4 h at 20 °C.

**Figure 3:**
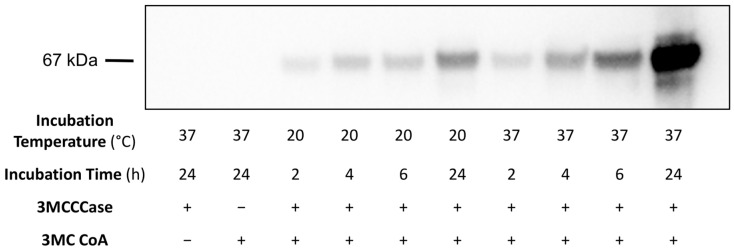
Effect of incubation time on *trans*-3MGC CoA-dependent acylation of BSA. A 3MCCCase assay was conducted and filtered to remove 3MCCCase. BSA (0.5 mg/mL) was added to the filtrate, and sample aliquots were then incubated for 2, 4, 6, and 24 h at either 20 °C or 37 °C. For control incubations, the 3MCCCase assay was conducted in the absence of either 3MC CoA substrate or 3MCCCase enzyme. Following incubation, each sample was probed for 3MGCylated BSA using α-3MGC IgG immunoblot analysis. The immunoblot shown is representative of an experiment performed on three separate occasions.

To examine the effect of 3MCCCase assay filtrate incubation time on 3MGCylated BSA signal intensity, a 3MCCCase assay filtrate was prepared, BSA was added, and aliquots incubated for 2, 4, 6, and 24 h at 20 °C and 37 °C, respectively. After the indicated incubation time intervals, sample aliquots were analyzed using α-3MGC immunoblot (Figure 3). In control incubations lacking either 3MCCCase or 3MC CoA, no 3MGCylated BSA signal was detected. When enzyme and substrate were present, however, incubations at 20 °C gave rise to a positive correlation between incubation time and immunoblot signal intensity. This trend also held for incubations conducted at 37 °C. Furthermore, at every time point tested, immunoblot signal intensity was stronger in samples incubated at 37 °C versus those incubated at 20 °C.

### 3.2. Anhydride Formation Studies

**Figure 4:**
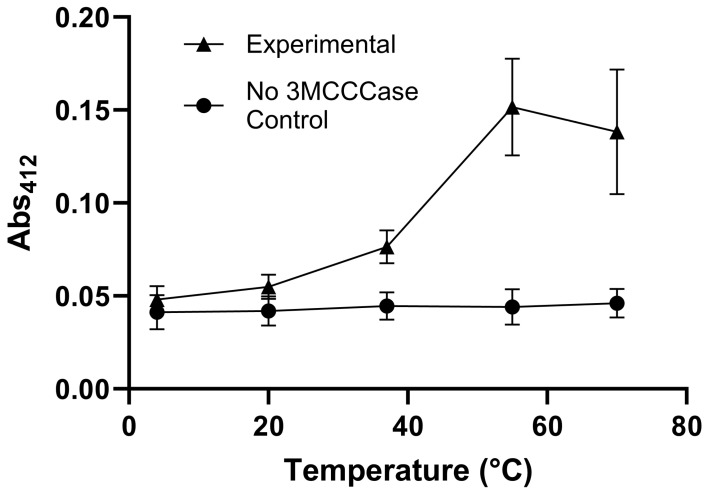
Effect of incubation temperature on *trans*-3MGC CoA conversion to *cis*-3MGC anhydride. A 3MCCCase assay filtrate and a control filtrate (containing all reaction components except 3MCCCase) were generated. To promote non-enzymatic intramolecular cyclization of *trans*-3MGC CoA, sample aliquots were incubated for a further 4 h at temperatures including 4, 20, 37, 55, and 70 °C. Following incubation, samples were returned to room temperature, DTNB was added, and sample absorbance was measured at 412 nm. The values reported are the mean ± standard deviation (n = 3) of three independent experiments.

In the non-enzymatic chemical reaction sequence depicted in Figure 1, *trans*-3MGC CoA isomerization to *cis*-3MGC CoA occurs prior to intramolecular cyclization to form 3MGC anhydride and free CoA. To investigate this intramolecular cyclization reaction, assays were performed to measure free CoA thiol content, a product of this reaction. A 3MCCCase assay filtrate was prepared, along with a control filtrate that contained all reaction components but no 3MCCCase. To study non-enzymatic intramolecular cyclization, filtrate aliquots were incubated for a further 4 h at indicated temperatures (Figure 4). Following incubation, samples were adjusted to room temperature, DTNB was added, and sample absorbance was measured at 412 nm. When 3MCCCase was omitted (control filtrate), sample free thiol levels remained low (near background levels) at every filtrate incubation temperature examined. On the other hand, in filtrate samples from 3MCCCase-containing assays, sample absorbance at 412 nm increased with increasing incubation temperature between 4 °C to 55 °C, consistent with temperature-induced 3MGC anhydride formation. When incubations were conducted at 70 °C, however, sample absorbance at 412 nm was similar to that observed at 55 °C, indicating that equivalent levels of free CoA thiol were generated in both samples.

### 3.3. Effect of Incubation Temperature on cis-3MGC Anhydride Hydrolysis

**Figure 5:**
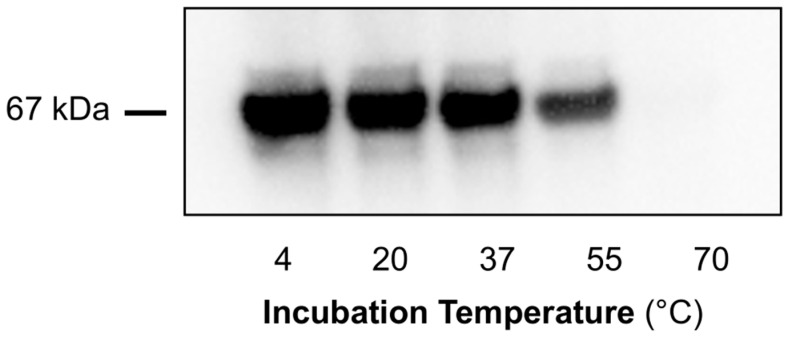
Effect of incubation temperature on hydrolysis of *cis*-3MGC anhydride. Aliquots of a 275 µM solution of 3MGC anhydride in 50 mM HEPES, pH 8.0, 20 mM MgCl_2_, and 20 mM KCl were incubated at 4, 20, 37, 55, and 70 °C, respectively, for 4 h. Following incubation, the samples were cooled, BSA was added to 0.5 mg/mL, and the samples were incubated for a further 1 h at 20 °C. After incubation with BSA, an aliquot of each sample was analyzed using α-3MGC IgG immunoblot to assess 3MGCylated BSA signal intensity. The immunoblot shown is representative of an experiment performed on three separate occasions.

To further investigate the sequence of the non-enzymatic reactions of *trans*-3MGC CoA, the effect of pre-incubation temperature on *cis*-3MGC anhydride-dependent acylation of BSA was studied. Aliquots of a 275 µM solution of *cis*-3MGC anhydride (obtained from a commercial vendor) were incubated for 4 h at the temperatures indicated (Figure 5). Following incubation, samples were cooled, BSA was added (0.5 mg/mL), and the samples were incubated for a further 1 h at 20 °C. Following incubation with BSA, an aliquot of each sample was analyzed using α-3MGC IgG immunoblot to assess 3MGCylated BSA signal intensity. As the temperature of *cis*-3MGC anhydride pre-incubations increased, a decrease in 3MGCylated BSA signal intensity was observed. For example, when *cis*-3MGC anhydride was pre-incubated at 4 °C for 4 h prior to the addition of BSA and incubated for a further 1 h at 20 °C, 3MGCylated BSA signal intensity was strong. However, when *cis*-3MGC anhydride was pre-incubated at higher temperatures (20 °C to 55 °C) prior to BSA addition and further incubation at 20 °C, BSA 3MGCylation signal intensity was reduced as a function of increasing pre-incubation temperature. These data indicate that *cis*-3MGC anhydride is more stable at 4 °C than it is when exposed to higher temperatures. Most likely, during the pre-incubation period, 3MGC anhydride undergoes temperature-dependent hydrolysis, yielding 3MGC acid, which is not capable of acylating substrate proteins. At 70 °C, no 3MGCylated BSA was detected, consistent with complete hydrolysis of 3MGC anhydride during the pre-incubation period.

### 3.4. Effect of AUH on trans-3MGC CoA-Dependent Acylation of BSA

**Figure 6:**
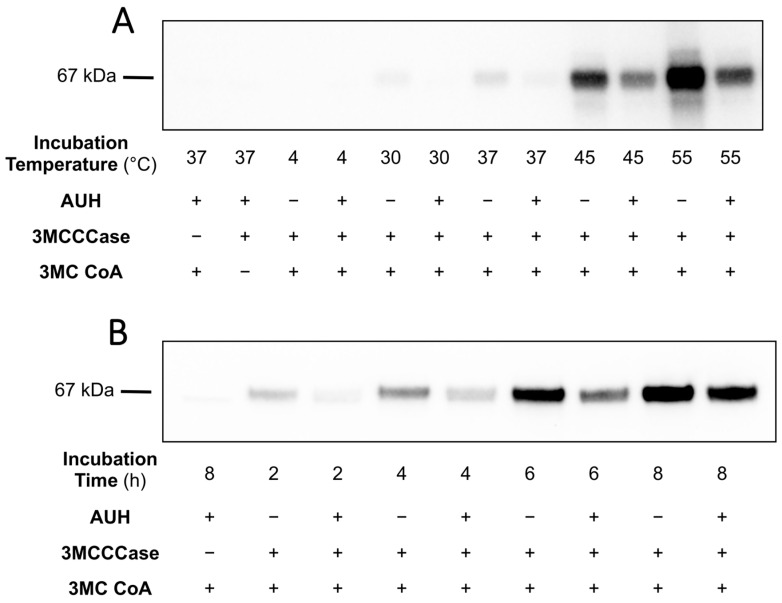
Effect of AUH on *trans*-3MGC CoA-dependent 3MGCylation of BSA. (**A**) 3MCCCase assays were conducted as described in Materials and Methods in the presence and absence of AUH (2 µg per 100 µL). BSA (0.5 mg/mL) was added, and aliquots were incubated at 4, 30, 37, 45, and 55 °C for 5 h. Following incubation, aliquots of each sample were subjected to α-3MGC IgG immunoblot analysis. Control incubations lacking enzyme or 3MC CoA were conducted in parallel. (**B**) As in Panel A, except aliquots were incubated at 37 °C for 2, 4, 6, and 8 h. Following incubation, aliquots of each sample were subjected to α-3MGC IgG immunoblot analysis. The immunoblots shown in Panels A and B are representative of experiments performed on three separate occasions.

As described above, *trans*-3MGC CoA undergoes a time- and temperature-dependent non-enzymatic chemical reaction sequence that results in protein 3MGCylation. To investigate the ability of AUH to attenuate this process, a 3MCCCase assay mixture containing BSA was incubated in the absence or presence of AUH. Individual samples were incubated at specified temperatures for 5 h. Following incubation, samples were subjected to α-3MGC IgG immunoblot analysis. The data revealed that in incubations conducted in the presence of AUH, α-3MGC IgG immunoblot signal intensity was attenuated across all temperatures examined as compared to control incubations lacking AUH (Figure 6A). Moreover, a positive correlation was observed between incubation temperature and α-3MGC IgG immunoblot signal intensity in both the presence and absence of AUH.

In a separate experiment, a 3MCCCase assay mixture containing BSA was incubated in the absence or presence of AUH. Samples were then incubated at 37 °C for 2, 4, 6, and 8 h, followed by α-3MGC IgG immunoblot detection. The data showed that the inclusion of AUH in the 3MCCCase assay mix attenuates 3MGCylated BSA signal intensity at each time point (Figure 6B). Consistent with the results presented in Figure 3, a positive correlation was observed between incubation time and 3MGCylated BSA signal intensity in the absence and presence of AUH.

### 3.5. Protein 3MGCylation in Mitochondria Homogenates from Liver-Specific HMGCL KO and Wild-Type Mice

**Figure 7:**
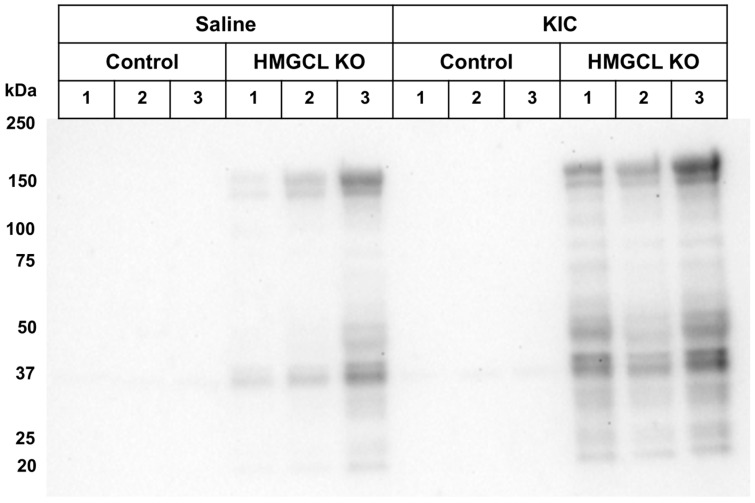
Effect of liver-specific HMGCL KO on protein 3MGCylation in mouse liver mitochondrial homogenates. HMGCL KO and control wild-type mice were administered saline or KIC by intraperitoneal injection, respectively. Following this, the animals were sacrificed, and liver tissue was harvested. Mitochondria were isolated from individual liver tissue sample homogenates, and equivalent protein aliquots of each sample were subjected to α-3MGC IgG immunoblot analysis. Each lane corresponds to an individual mouse liver sample.

To evaluate protein 3MGCylation in vivo, liver mitochondria were isolated from saline-treated wild-type mice and liver-specific HMGCL KO mice. Mitochondrial homogenates were also obtained from wild-type and liver-specific HMGCL KO mice that had received a load of the upstream leucine catabolic pathway intermediate, KIC. Homogenates from isolated liver mitochondria from each mouse group were subjected to α-3MGC IgG immunoblot analysis to assess the extent of protein 3MGCylation (Figure 7). In the case of wild-type mice administered either saline or KIC, immunoblot signal intensity was weak, indicating that few, if any, mitochondrial proteins are 3MGCylated in wild-type mice. By comparison, mitochondria homogenates derived from saline- and KIC-treated liver-specific KO mice contained numerous distinct bands positive for protein 3MGCylation. Of note, whereas the banding pattern was similar in saline- and KIC-loaded HMGCL KO mice, the α-3MGC IgG immunoblot signal intensity was stronger in KIC-treated HMGCL KO liver mitochondrial homogenates than in homogenates from saline-treated HMGCL KO mice.

## 4. Discussion

**Figure 8:**
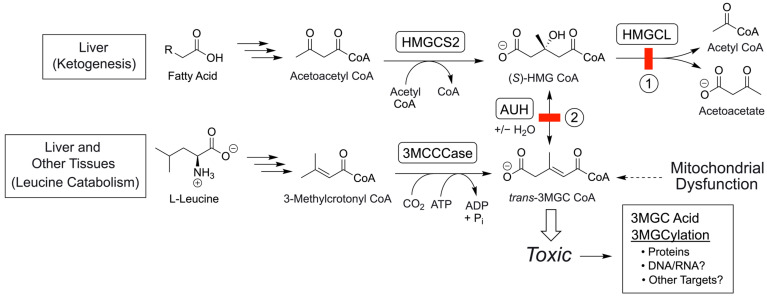
IEM-induced formation of toxic byproducts of *trans*-3MGC CoA. In primary 3MGC aciduria, a deficiency in HMGCL causes *trans*-3MGC CoA levels to rise. In this disorder, two distinct biochemical pathways are affected: ketogenesis and leucine catabolism (see pathway block #1, depicted with a red box). Since HMG CoA cannot be metabolized to acetoacetate and acetyl CoA in this case, it is instead dehydrated to *trans*-3MGC CoA via AUH, which then generates toxic metabolites via a series of non-enzymatic chemical reactions (see Figure 1). In a similar but distinct manner, a deficiency in AUH (pathway block #2 depicted with a red box) also leads to a buildup of *trans*-3MGC CoA but only from leucine degradation (the ketogenesis pathway is unaffected in this IEM). In secondary 3MGC aciduria, labeled as “Mitochondrial Dysfunction” in this figure, whereas no leucine/ketogenesis pathway deficiencies exist (that is, both AUH and HMGCL are functional), various other IEMs (not shown) adversely affect mitochondrial energy metabolism, resulting in the diversion of acetyl CoA away from TCA cycle entry toward *trans*-3MGC CoA [22]. In each instance, as *trans*-3MGC CoA is formed, it is susceptible to the same series of non-enzymatic chemical reactions, resulting in the production of toxic byproducts, including 3MGC acid, protein 3MGCylation, and, conceivably, acylation of other amine-containing biomolecules in mitochondria. Note that although two red boxes are shown, only one or the other is deficient in any given scenario. HMGCS2 = HMG CoA Synthase 2.

3MGC aciduria is a characteristic phenotypic feature of over 20 IEMs [2]. In every case, the 3MGC carbon skeleton is derived from *trans*-3MGC CoA. Interestingly, however, two distinct categories of 3MGC aciduria exist (primary and secondary), which are distinguished by the metabolic route to *trans*-3MGC CoA [21]. In primary 3MGC aciduria, IEMs resulting in AUH or HMGCL deficiencies block leucine degradation, directly leading to a buildup of the upstream intermediate, *trans*-3MGC CoA (see Figure 1). Because HMGCL also functions in ketogenesis (liver and kidney tissues), when this enzyme is missing or deficient, ketone body biogenesis is also blocked. As seen in Figure 8, when HMGCL is deficient (IEM #1, see red box), HMG CoA accumulates with subsequent AUH-mediated dehydration of HMG CoA to *trans*-3MGC CoA and toxic byproduct formation (note that in this case, AUH is fully active). Thus, when HMGCL is deficient, there are two potential sources of *trans*-3MGC CoA: leucine catabolism and ketogenesis. IEMs in *AUH* (IEM #2) also lead to *trans*-3MGC CoA but only via the leucine degradation pathway.

In secondary 3MGC acidurias, HMGCL and AUH are fully functional. Instead, IEMs in discrete genes involved in mitochondrial energy metabolism are responsible for the phenotype [2]. These IEMs lead to one or more of the following: electron transport chain (ETC) dysfunction, defective cristae membrane integrity, decreased protein chaperone activity, cardiolipin abnormalities, or other mitochondrial malfunctions [22]. As a result, ETC activity decreases, and reduced cofactors (NADH and FADH_2_) are unable to transfer reducing equivalents to Complex I/II, leading to a buildup of NADH/FADH_2_ in the matrix space. This leads to the inhibition of TCA cycle enzymes that produce these reduced cofactors. As a result, TCA cycle activity slows and acetyl CoA levels rise. Under these conditions, the matrix enzyme, acetoacetyl CoA (T2) thiolase, functions in reverse, condensing two acetyl CoA to form acetoacetyl CoA. Subsequently, HMG CoA synthase 2-mediated condensation of acetoacetyl CoA with a third acetyl CoA produces HMG CoA, which, in extrahepatic tissue mitochondria, has few options. Although extrahepatic tissue mitochondria express small amounts of HMGCL, their expression of succinyl CoA:3-oxoacid CoA transferase [23], combined with impaired ETC function, can potentially lead to the generation of more HMG CoA. Over time, however, some portion of the HMG CoA pool is dehydrated by AUH to *trans*-3MGC CoA.

In most metabolic enzyme deficiencies, mitochondria possess strategies to prevent CoA depletion as a result of the buildup of acyl CoA intermediates. These strategies include the carnitine acyltransferase-dependent conversion of acyl CoAs to the corresponding acylcarnitine and free CoA [24], as well as acyl CoA thioesterase-mediated CoA hydrolysis to yield the corresponding organic acid [25]. To date, however, there is no evidence that either of these mechanisms functions to prevent a deleterious loss of free CoA in the case of *trans*-3MGC CoA. This may be due to the fact that *trans*-3MGC CoA is an inherently labile compound that undergoes non-enzymatic isomerization to *cis*-3MGC CoA under physiological conditions. Unlike *trans*-3MGC CoA, however, *cis*-3MGC CoA is structurally poised to undergo intramolecular cyclization with loss of CoA, forming *cis*-3MGC anhydride. This cyclic anhydride is reactive, with at least two potential fates, including hydrolysis to form *cis*-3MGC acid or acylation of protein lysine side chain amino groups (i.e., 3MGCylation). It must also be considered that other biomolecules in the cell that possess a primary amine functional group with a pKa above 8 are susceptible to 3MGCylation. For example, DNA bases or phosphatidylethanolamine could conceivably be acylated by 3MGC anhydride, with potentially severe toxic consequences. As *trans*-3MGC CoA levels are depleted via this non-enzymatic chemical reaction sequence, a chemical sink is created that, in the case of HMGCL deficiency, leads to increased AUH-dependent dehydration of HMG CoA [8]. In IEMs causative of either primary or secondary 3MGC aciduria, the same chemical sink exists, wherein the net effect is the preservation of the mitochondrial pool of free CoA [7]. It is noteworthy that, in secondary 3MGC aciduria, much lower amounts of 3MGC acid appear in the urine as compared to primary 3MGC aciduria, consistent with the different metabolic origins of *trans*-3MGC CoA [1,26].

From a chemical standpoint, an important question relates to why *trans*-3MGC CoA is susceptible to this sequence of non-enzymatic chemical reactions. Whereas no previous studies have examined the chemical properties of *trans*-3MGC CoA in detail, Jones et al. [27] investigated the isomerization potential of *trans*-3MGC acid. These authors propose that the methyl group on carbon 3 of *trans*-3MGC acid plays a key role by forming a relatively stable tertiary allylic carbocation at carbon 3 upon transient double bond migration from the 2–3 position to the 1–2 position. When this occurs, free rotation around the resulting sigma bond between carbons 2 and 3 is possible. Upon reversion of the double bond back to the 2–3 position, depending on its bond rotation status, either a *cis*- or *trans*-configuration will result. Compared with *trans*-3MGC acid, *trans*-3MGC CoA is likely more susceptible to isomerization and, unlike the acid, *cis*-3MGC CoA is susceptible to intramolecular cyclization followed by hydrolysis or acylation. As discussed above, the *cis*-configuration of 3MGC CoA positions the terminal carboxylic moiety (carbon 5) adjacent to the CoA thioester on carbon 1. Subsequent reactions between these functional groups result in the loss of H_2_O and CoA and cyclization, forming *cis*-3MGC anhydride. The anhydride is chemically reactive, and given the protein-rich environment of the mitochondrial matrix, it reacts with lysine side chain amino groups to acylate nearby proteins. Alternatively, *cis*-3MGC anhydride can undergo spontaneous hydrolysis to yield 3MGC acid.

In the present study, various aspects of *trans*-3MGC CoA reactivity have been investigated. Initial studies were performed to identify conditions wherein *trans*-3MGC CoA could be produced yet remain stable. An in vitro enzyme assay involving the 3MCCCase-mediated carboxylation of the unsaturated acyl CoA substrate, 3MC CoA, was employed. By conducting this assay at 20 °C, although *trans*-3MGC CoA was generated, the occurrence of unwanted spontaneous chemical reactions was minimized. Removal of 3MCCCase from the reaction mixture through spin filtration effectively halts the reaction so that no more *trans*-3MGC CoA is produced. The resulting 3MCCCase assay filtrate was then used to investigate *trans*-3MGC CoA-dependent acylation of BSA, a readout of 3MGC anhydride formation. By using this assay, evidence was obtained that α-3MGC IgG immunoblot signal intensity increased as a function of time and temperature in filtrate incubations with BSA. Moreover, studies with the thiol reactive reagent, DTNB, provided independent evidence that spontaneous chemical reactions of *trans*-3MGC CoA proceed through 3MGC anhydride via an intramolecular cyclization reaction that is temperature-dependent. The α-3MGC IgG immunoblot detection method employed was shown to represent a reliable readout of *trans*-3MGC CoA chemical reactivity, and this approach was also employed in experiments designed to show that *trans*-3MGC CoA hydratase (AUH) activity leads to the attenuation of immunoblot signal intensity in 3MCCCase assay incubations with BSA. This result is achieved by diverting some portion of the *trans*-3MGC CoA pool to HMG CoA. Based on this result, it is anticipated that if HMGCL activity were also present, an even greater degree of signal attenuation would be observed because acetoacetate and acetyl CoA would be the final products rather than HMG CoA, which can be dehydrated back to *trans*-3MGC CoA.

Following the characterization of protein 3MGCylation in vitro, studies employing liver-specific HMGCL KO and wild-type mice were conducted. Isolated liver mitochondria homogenates were prepared and used to investigate protein 3MGCylation in vivo. Relative protein 3MGCylation levels were much higher in liver-specific HMGCL KO mouse liver mitochondrial samples compared with the corresponding WT mouse samples. Moreover, KIC loading led to increased protein 3MGCylation levels in liver-specific HMGCL KO mouse liver mitochondria as compared to saline-treated liver-specific HMGCL KO mouse mitochondrial samples. This result confirms that KIC treatment has a positive effect on mitochondrial protein 3MGCylation in liver-specific HMGCL KO mouse mitochondria. The results obtained support the concept that KIC feeds into the leucine degradation pathway, causing an increased buildup of the pathway intermediate, *trans*-3MGC CoA.

## 5. Conclusions

In conclusion, the results presented provide evidence in support of the concept that, in a variety of IEMs that affect leucine metabolism or ketogenesis (primary 3MGC aciduria) or ETC function (secondary 3MGC aciduria), as *trans*-3MGC CoA accumulates, it undergoes a non-enzymatic chemical reaction sequence that preserves the free CoA pool in mitochondria while yielding 3MGC acid and 3MGCylated proteins. Future studies are required to identify the proteins that are 3MGCylated and determine whether specific lysine residues are targeted or if 3MGC anhydride reaction with protein lysine side chains is random. Two additional factors of interest include the potentially deleterious effects of 3MGCylation on protein function, as well as the relative efficiency with which the NAD^+^-dependent deacylase, sirtuin 4 [10], is able to remove 3MGC moieties from mitochondrial proteins. An important future area of research will be to examine the extent to which the accumulation of *trans*-3MGC CoA leads to covalent acylation of non-protein amine-containing substrates such as DNA, RNA, phosphatidylethanolamine, and/or amino sugars. Answers to these questions will likely provide novel insight into disease phenotypes associated with different forms of 3MGC aciduria.

## Data Availability

The data presented in this study are available within the article.
